# In the Words of the Medical Tourist: An Analysis of Internet Narratives by Health Travelers to Turkey

**DOI:** 10.2196/jmir.2694

**Published:** 2014-02-06

**Authors:** Margaret E Ozan-Rafferty, James A Johnson, Gulzar H Shah, Attila Kursun

**Affiliations:** ^1^Dignity Health, Nevada MarketSt. Rose Dominican HospitalsHenderson, NVUnited States; ^2^Central Michigan UniveristyHerbert H and Grace A. Dow College of Health ProfessionsMount Pleasant, MIUnited States; ^3^Georgia Southern UniversityJiann-Ping Hsu College of Public HealthStatesboro, GAUnited States; ^4^JADA Healthcare Services, LLCKing of Prussia, PAUnited States

**Keywords:** medical tourism, Turkey, travel blogging, personal narratives, qualitative research, patient satisfaction, delivery of health care, globalization, social media internationality

## Abstract

**Background:**

Patients regularly travel to the West for advanced medical care, but now the trend is also shifting in the opposite direction. Many people from Western countries now seek care outside of their country. This phenomenon has been labeled medical tourism or health travel. Information regarding health travelers’ actual outcomes, experiences, and perceptions is lacking or insufficient. However, advanced Internet technology and apps provide information on medical tourism and are a vehicle for patients to share their experiences. Turkey has a large number of internationally accredited hospitals, is a top tourism destination, and is positioning itself to attract international patients.

**Objective:**

The objective of this research was to identify the important individual characteristics of health travelers, outline the push and pull factors for seeking health care in Turkey, identify satisfaction with the outcomes and the results of these individuals’ treatments, and note positive and negative factors influencing their perceptions and overall experiences about patients’ health travel.

**Methods:**

This research uses qualitative data from Internet narratives of medical tourists to Turkey. Ethical considerations of using Internet narratives were reviewed. Narratives for analysis were obtained by using the Google search engine and using multiple search terms to obtain publicly posted blogs and discussion board postings of health travelers via purposeful sampling. Narratives were included if they were written in English, described travel to Turkey for health care, and were publicly accessible. Exclusion criteria included narratives that were on medical tourism facilitator or provider promotional websites, not in English, and did not describe an experience of a medical tourist. Medical tourists’ written words were analyzed in an iterative analytic process using narrative analysis theory principles. Three stages of coding (open, axial, and selective) were conducted to identify characteristics and themes using qualitative analysis software.

**Results:**

The narrative posts of 36 individuals undergoing 47 procedures who traveled to Turkey for medical care between 2007 and 2012 were analyzed. The narratives came from 13 countries, not including the narratives for which patient origin could not be determined. Travelers were predominantly from Europe (16/36, 44%) and North America (10/36, 28%). Factors driving travelers away from their home country (push factors) were cost and lack of treatment options or insufficient insurance coverage in their home country. Leading factors attracting patients to destination (pull factors) were lower costs, physician’s expertise and responsiveness, and familiarity or interest in Turkey. Health travelers to Turkey were generally satisfied with the outcomes of their procedures and care provided by their physicians, many noting intent to return. Communication challenges, food, transportation, and gaps in customer service emerged as key areas for improvement.

**Conclusions:**

This analysis provides an understanding of the insights of medical tourists through the words of actual health travelers. This nonintrusive methodology provides candid insights of common themes of health travelers and may be applied to study other patient experiences. The findings of this research expands the body of knowledge in medical tourism and serves as a platform for further qualitative and quantitative research on health travelers’ experiences.

## Introduction

### Background

Historically, patients have received health care in the country of their residence, but more recently, medical-related travel to other countries has substantially transformed this traditional mode of health care [1]. Although travel to receive medical care in another country is bidirectional, many developing countries are promoting their health care services to attract foreign patients [1-4].This phenomenon has been accelerated by increasing globalization, improved bilateral trade [5,6], patients’ unmet health care needs, and increasing cost of services within the United States [7,8]. Travel for health care today has many forms. Although cosmetic procedures are popular, health travelers also seek open-heart surgery, hip or knee replacement [9-11], wellness treatments [12], and procedures yet to be approved in their home country [13]. Many estimates indicate medical tourism, also known as health travel [14], has significant economic potential worldwide [10,15], and many countries are positioning themselves to be providers of care and service for international patients [11,16-20].

The theoretical explanation of health travel may benefit from theories explaining human migration, although the former is different from migration in that migration involves somewhat permanent geographic relocation. Consistent with the push-pull theory of migration [21], moving from one country to another is typically influenced by factors that push individuals away from their country of origin, driving people to leave home, and factors that pull or attract people to a new country [22]. In their scoping review of the health tourists’ experiences, Crooks et al [23] noted that both push and pull factors contributed to patient decision making when considering traveling for care.

### Purpose of the Study

Information on patient’s experiences of their care is a foundational aspect of health care quality [24]. Although surveys and interviews provide insights into care provided, over the past several years patients have begun to share their experiences on the Internet in the form of blogs, discussion posts, and tweets resulting in large amounts of data which may augment formal survey approaches [25] To date, there are few studies in the medical tourism literature that evaluate health travelers’ experiences with care in another country [23]. Focusing on the narrative experiences of health travelers also provides feedback into the emotions associated with seeking care in an unfamiliar environment [26]. Limited accurate national data on the number, type, and impact of individuals who travel for health care have led to potentially inflated claims of industry growth [27] and made it difficult to address policy and public health needs of health travelers [28]. The majority of the published studies have focused on scoping reviews of the existing literature and identify research gaps [4,5,11,29,30]. Lack of detailed information on the common experiences of health travelers may result in inaccurate assumptions and misleading conclusions regarding their experiences and outcomes.

After an evaluation of the literature on health travel, Turkey was selected for this study because it has a large number (n=47) of internationally accredited health care providers [31] and is the world’s sixth top tourism destination by arrivals [32]. Turkey boasts a geographic location attractive to European, Middle Eastern, African, and Asian health travelers [33], and is implementing multiple initiatives to make health and wellness care in Turkey attractive to international patients [34-36]. Although reports of the volume of health travelers vary, between 2008 and 2011 the number more than doubled according to the Directorate General of Health Services Department of Health Tourism, making the quality of health care in Turkey increasingly important on a global scale [34,37].

This qualitative narrative study explores the online narratives from health travelers to Turkey and describes the themes of their experiences. Qualitative research studies can capture an in-depth understanding of an issue [38]. Narrative research “begins with the experiences as expressed in lived and told stories of individuals” [38]. Although Internet and online documentation are relatively new phenomena, narrative analysis of online content has been conducted in several disciplines, including health care and travel [39-43].

It is expected that findings from this research will assist in decision making for patients considering health travel in the future, assist countries with their marketing and positioning to health travelers, and serve as a resource for hospitals wanting to recruit and retain staff to serve a global patient base. In addition, this research may strengthen health administration education by providing insights into the phenomenon of health travel.

### Research Questions

The overarching question answered in this research is “What can we learn about health travelers to Turkey through analysis of their online narratives?” Additional subquestions included:

1. What are the important characteristics of health travelers who write online narratives about their experiences in Turkey?

2. What are the leading factors associated with the health travelers’ country of origin that pushed them to seek health care in another country?

3. What are the leading factors associated with the destination country, Turkey, which pulled these health travelers to seek health care?

4. What can be derived from the narratives regarding travelers’ satisfaction with the outcome and the result of their treatment they received in Turkey?

5. What are some positive and negative factors influencing health travelers’ perceptions and overall experiences about their health travel to Turkey?

## Methods

### Search Strategy

Publicly available narratives were considered for this research after the ethical considerations of using online narratives were reviewed [44-49]. After receiving approval from the Central Michigan University Institutional Review Board, the narratives for analysis were obtained by using the Google search engine. Multiple search terms were used to obtain as many publicly posted narratives of health travelers to Turkey via purposeful sampling [50] during October 2012. The search utilized a range of terms, including health travel, medical tourism, surgery, and wellness travel. Clinical procedures used in the search were determined based on reviewing sites that promote health travel as well as findings from literature review and terms noted in selected posts, such as “I had surgery in Turkey.”

The search was further refined by using Google’s advanced search process in which the search terms were limited to discussions and blogs. Each result from the first 10 pages of these searches was reviewed to meet the criteria of: (1) a narrative written in English because all researchers were fluent in this language, (2) a first-person narrative written by an individual who underwent treatment and/or their significant other/partner on the trip, (3) included a description of the type of procedure, (4) a narrative that included a personal actual experience of health travel to Turkey, and (5) a narrative that was publicly available and did not require a password or discussion board membership to read. The authors reviewed all narratives to ensure promotional narratives found on health travel facilitator, promoter, or health care provider sites were excluded to avoid potential bias [28]. All narratives selected noted the name of the procedure performed and mentioned that this procedure was performed in Turkey.

### Search Results

**Figure 1:**
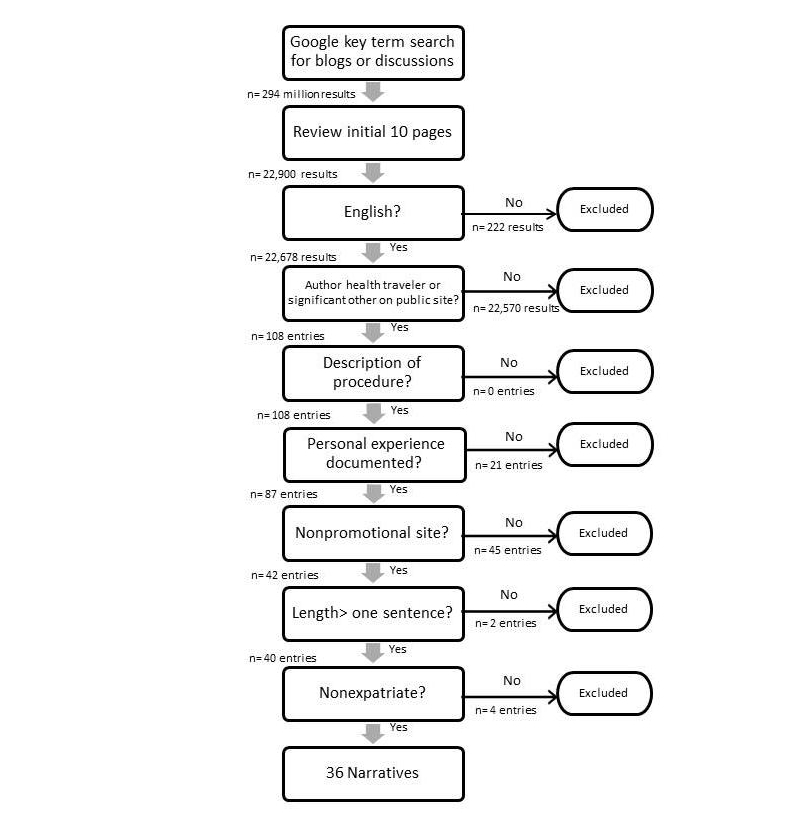
Steps in the narrative search process and findings.

The initial Internet searches using 229 search terms resulted in 294,125,697 entries including those in languages other than English. Figure 1 denotes the narrative search process findings. Using the inclusion and exclusion criteria and taking a flexible approach to defining a narrative, both stories of the entire experience as well as those that were shorter in length highlighting a specific part of the health travel experience were included [41]. Narratives consisted of some discussion board posts of a few paragraphs on a single day to blogs that consisted of posts spanning several months with multiple entries. Only posts and blogs related to the topic of the author’s health travel were included in the research.

After extensive searching, an initial 40 narratives were found that met the criteria. After a secondary review by the authors, 4 narratives were eliminated because further review indicated they may have been written by expatriates living in Turkey. A total of 36 narratives were deemed usable for the research, consisting of 23 message board or forum postings and 13 blogs.

### Coding Process

Posters’ written words were analyzed in an iterative analytic process by using narrative analysis theory principles to create primary (parent) and secondary (child) themes. The unit of reference was a sentence [51,52]. Initial open coding was conducted on all pages of the 36 final posts to identify common themes and create initial codes for the data [50]. Each narrative was carefully reviewed to identify themes, with a focus on the initial research question and subquestions. In addition, the content of each sentence was directly examined to identify positive and negative opinions [52]. A total of 41 initial themes were identified and memos were written to keep track of ideas and observations. Two researchers, the coauthors of this paper, individually reviewed 5 cases and compared their agreements/disagreements on the major themes to assure level of intercoder reliability. Percent agreement on the 41 themes was 95.

Axial coding, or a second review of the data, was then completed and the 36 individual narratives were entered into NVivo version 10 [53] where the initial codes and key concepts identified in the open coding were distilled and clustering of the initial themes occurred [50]. Relationships with the various initial themes were created and characteristics of each case, where available, were noted.

A final round of selective coding [50] was completed by scanning all the data and codes, reviewing all themes and creating major themes (parent) and subthemes (child), and reorganizing the themes. During this review, linkages between some themes and the elimination of other initial themes by consolidation of themes were completed. Narratives from the posters representing each major theme were selected and personal details were removed from the quotes.

## Results

### Health Travelers’ Characteristics

The subjects (ie, posters used in the analysis) were identified by country of origin as shown in Table 1.

As shown in Table 2, the posters traveled to Turkey for a myriad of procedures. The 36 posters went for a total of 47 procedures. Dental work was the most frequent secondary procedure mentioned by the posters.

Demographic information, such as gender and age, is provided in Table 3 as well as some information on the facility or locale of the treatment. Of those who noted the place of their treatment, Anadolu Medical Center was mentioned most frequently. Most posters (17/36, 47%) noted that they had their procedure completed in Istanbul, 7 (7/36, 19%) went to Izmir, and 2 (2/36, 6%) traveled to Ankara. In all, 9 posters (9/36, 25%) mentioned a medical tourism facilitator organization by name and 3 (3/36, 8%) posters noted that they used the same facilitator.

**Table 1:** Health travelers’ country of origin (N=36).

Country		Number of travelers
**North America (n=10, 28%)**		
	United States	8
	Canada	2
**Europe (n=16, 44%)**		
	United Kingdom	9
	Netherlands	2
	Finland	1
	Germany	1
	Ireland	1
	Macedonia	1
	Romania	1
**Middle East (n=2, 6%)**		
	Dubai	1
	Israel	1
Kenya (n=1, 3%)		1
Korea (n=1, 3%)		1
Undetermined (n=6, 16%)		6

**Table 2:** Health travelers’ primary and additional procedures performed in Turkey.

Procedure type	Procedure, n (%)		
	Primary procedure^a^ (n=36)	Additional procedure (n=11)	Total (n=47)
Hair transplant	12 (26)		12 (26)
Dental	2 (4)	3 (6)	5 (11)
Abdominoplasty	3 (6)	1 (2)	4 (9)
Breast enlargement	3 (6)	1 (2)	4 (9)
Lasik	4 (9)		4 (9)
In vitro fertilization	2 (4)		2 (4)
Liposuction		2 (4)	2 (4)
Nerve surgery	2 (4)		2 (4)
Rhinoplasty	2 (4)		2 (4)
Back surgery	1 (2)		1 (2)
Bariatric	1 (2)		1 (2)
Botox and fillers		1 (2)	1 (2)
Cancer treatment	1 (2)		1 (2)
Cyst removal		1 (2)	1 (2)
Face lift		1 (2)	1 (2)
Heart surgery	1 (2)		1 (2)
Labioplasty		1 (2)	1 (2)
Physical exam	1 (2)		1 (2)
Stem cell transplant	1 (2)		1 (2)

^a^Primary procedure is the procedure posters noted first in their narrative.

**Table 3:** Health travelers’ date of initial narrative, demographics, location of treatment, date of treatment, facilitator facility, and accompanying person.

Author	First post	Gender	Age	City	Date of treatment	Facilitator	Facility	Accompanying person
#1	7/22/10	M	30s	Izmir	5/1/10	—	—	—
#2	2/1/09	—	—	Istanbul	—	Blue Med travel	—	—
#3	4/1/12	M	—	Istanbul	—	—	Este of Turkey	—
#4	6/3/12	F	—	Izmir	6/1/12	—	Kent	—
#5	8/12/12	F	60s	Istanbul	10/12/12	Comfort Zone	—	—
#6	2/16/09	F	—	—	—	—	—	Spouse
#7	2/26/11	F	30s	Istanbul	3/12/11	World Med assist	Anadolu Hospital	Partner
#8	2/14/07	F	—	Izmir	—	Revitalize in Turkey	—	—
#9	9/13/12	F	—	—	—	—	Dunya Eye	Other referenced
#10	3/14/10	M	—	Ankara	2/22/10	—	Dr. Keser Clinic	—
#11	4/1/12	—	—	—	—	—	Este of Turkey	—
#12	6/7/11	M	50s	Istanbul	5/25/11	—	Transmed	—
#13	4/9/09	M	50s	Istanbul	5/1/09	World Med assist	Anadolu Hospital	—
#14	2/26/12	F	30s	Istanbul	5/1/12	IVF vacation center	Anadolu Hospital	Spouse
#15	5/22/11	F	—	Altinkum	—	—	—	Spouse
#16	N/A	M	30s	—	—	—	Hairana	—
#17	1/12/12	F	—	Izmir	12/12/11	Revitalize in Turkey	—	—
#18	1/14/09	F	—	—	1/2/09	—	—	—
#19	6/14/12	M	20s	Istanbul	9/1/11	—	Transmed	—
#20	10/17/12	M	30s	Istanbul	1/1/12	—	New Age Clinic	—
#21	5/7/08	M	20s	—	5/8/08	—	—	—
#22	10/9/07	M	—	Istanbul	8/9/07	—	Transmed	—
#23	8/30/12	M	20s	Ankara	8/1/12	—	Dr. Keser Clinic	—
#24	12/22/09	F	—	Istanbul	—	—	—	—
#25	7/4/12	F	40s	—	10/3/12	—	—	—
#26	4/8/11	M	40s	Izmir	5/19/11	—	—	Other family member
#27	9/24/12	F	—	—	5/1/12	—	—	—
#28	5/25/12	M	30s	Istanbul	5/12/12	—	Acibadem Hospital	—
#29	2/27/09	M	40s	Istanbul	4/1/09	World Med assist	Anadolu Hospital	Spouse
#30	5/12/12	M	30s	Istanbul	—	—	Hedz International	—
#31	1/29/12	F	60s	Istanbul	1/25/12	—	—	—
#32	5/3/12	F	—	—	9/1/11	—	—	—
#33	9/10/12	F	30s	Istanbul	10/12/12	—	Memorial Hospital	Spouse
#34	4/12/11	—	—	Izmir	6/19/12	—	—	—
#35	4/28/12	F	40s	Izmir	4/10/12	—	—	—
#36	2/2/12	F	—	Istanbul	—	Blue Med	—	—

### Decision Analysis

#### Push Factors

The subjects of this study noted a variety of reasons, also known as *push factors*, for their decision to seek care outside of their home country. As noted in Table 4, push factors included lack of available treatment in their home country and financial reasons based on price or lack of insurance.

**Table 4:** Push and pull factors for patients who traveled to Turkey for medical procedures.

Factors		Discussion
**Push factors**		
	Lack of treatment options in country of origin (3/36, 8%)	Several posters sought out treatment that was not available in their home country. These procedures included Cyber Knife treatment, stem cell transplants, and pudendal nerve surgery. These posters wrote about their disappointment with their inability to procure care in their own country.
	Cost (22/36, 61%)	Posters note the price of procedures in their home country as rationale for seeking care in another country. Although only a few noted actual cost comparisons that included figures, many shared that they investigated costs abroad and noted that not only was the price of the procedure less than at home, many organizations offered packages which were all-inclusive and provided transportation, meals, and accommodations.
	Lack of or insufficient insurance coverage (5/36, 14%)	The 3 US posters who went to Turkey for noncosmetic procedures, all noted that their surgeries were not covered by their insurance and, in one case, the patient did not have insurance. Two of these individuals had procedures which they noted were “experimental” in the United States, the other had heart surgery, but would have had to pay out of pocket because he lacked medical insurance coverage. One of the posters who traveled from the Netherlands for eye surgery noted that her insurance would only cover part of the cost of the procedure.
**Pull factors**		
	Comparative value (24/36, 67%)	Posters shared their approach to learning more about Turkey and their procedures and generally noted that their ability to compare overall value of care (as defined by Porter and Teisberg [54]) available through alternatives options assisted with their decision making. The research performed by the posters was often detailed and time consuming. Many posters shared the options they investigated during their research process. The ability to connect with others who traveled to Turkey for information and support was also a component of some posters pretravel research.
	Physician expertise and responsiveness (21/36, 58%)	Many posters mentioned the qualifications of the physicians as a factor that attracted them to care in Turkey. Some noted the background of the physician. One poster noted that it was the physician qualifications above costs that attracted them to Turkey. Other posters noted the communication or responsiveness of the physicians directly that attracted them to health travel to Turkey.
	Familiarity or interest in Turkey (5/36, 14%)	Some of the posters noted that they had lived in Turkey, worked in Turkey, or been on vacation or honeymooned in Turkey and others noted an interest in exploring Turkey. Two of the posters noted they were of Turkish descent.
	Availability of a health travel facilitator (13/36, 36%)	Some of the posters reached out to health travel facilitators as part of their process. Posters who mentioned using a health travel facilitator, someone typically on the ground in Turkey who serves in a sales consultative role, noted that the facilitators were responsive to their initial and online requests for information and assisted in the process of arranging treatments, transferring medical records, travel, and accommodations. Many of the facilitators were mentioned by name by the posters and several mentioned how quickly the facilitators responded to requests for information.
	Price (23/36, 64%)	Many of the posters noted that they were attracted to Turkey for health travel because of the perceived reasonable cost of travel, visa entry, and price of treatment. The all-inclusive packages mentioned by the posters often included transportation, mobile phone support, meals, and accommodations in addition to the cost of the medical treatment.

#### Pull Factors

Table 4 also outlines a number of factors that influenced the posters’ decisions to seek health care in Turkey. Many of the posters noted researching their options, primarily on the Internet, and then either initiated contact with a provider/physician in Turkey directly or utilized a health travel facilitator for assistance. Price and a familiarity with Turkey were also factors that attracted posters to Turkey.

### Satisfaction With Outcomes and Results of Treatment

Most posters (27/36, 75%) noted that they were satisfied with the outcome of their medical care in Turkey. Two hair transplant posters wrote negative reviews on a discussion board. One poster noted she was awaiting improvement in her nerve after surgery and the blogger who had end-stage cancer treatment died several months after treatment in Turkey.

Some posters (6/36, 17%) mentioned the impact that the procedure had on their lives. One of the posters who had been unable to stand up straight before his surgery wrote about the experience of being able to look up after his 12-hour surgery in Izmir:

On Sunday 22 May...my doctor asked if I was up to waking up from my bed and take a walk. What? Was he serious? I agreed and with the help of the nurses, I woke up from my bed with all the tubes hanging out. Boy did it feel weird taking my first steps. Felt like my back was in a vice. Walked to the windows and after so many years I was able to look up. You can’t believe the feeling that I started to cry, yes, at my age but hell yes I cried with joy and victory.

Very specific clinical outcome data including medical reports were noted as methods posters used to share the outcomes of their procedures. Several posters (7/36, 19%) offered to avail themselves via their personal email to anyone who needed additional information on their treatment and outcomes: “It has been 10 months since my last surgery and I have already had satisfying results that I would like to share with my fellow men who have been suffering from the same problem and hopefully I can be helpful as much as I can.”

Several posters (4/36 11%) who were satisfied with their outcomes also mentioned an intent to return for additional procedures: “We will be going back there later this year as my husband still needs some work carried out to finish off his implants.” Some of the posters who were less than satisfied with the outcome of their treatment also noted a desire to return in their posts as well. One couple who blogged about their failed in vitro fertilization attempts remained positive about their experiences in Turkey despite the negative outcome. Others were very direct in sharing their dissatisfaction and one even labeled their post “My disastrous journey with [facility]:”

This surgery with [facility] has made my life a living hell. I am not depressed but emotionally devastated.

One poster who wrote about being satisfied with her results did mention an infection after her procedure, but she noted that she blamed herself for not strictly following her postoperative instructions. The wife of a poster made the final entry on his blog announcing his death several months after end-stage cancer treatment.

### Perceptions and Overall Experiences

#### Positive Factors

##### Overview

The narratives included feedback on physicians, the facilitators, and the staff who cared for them or their loved one. Posters also wrote about the value they perceived, their perceptions of Turkey and the facilities where they received their care, and some gave details about their follow-up care.

##### Impression of Turkey

Most posters (10/11, 91%) who wrote about Turkey used favorable terms in their overall impressions. A couple posters compared Istanbul to other cities or their own country. Posters also noted their impressions of the people they met while in Turkey: “Turkish people are really friendly and were always willing to help out.”

Approximately half of the posters (6/11, 55%) who wrote about Turkey mentioned taking or making the time to see the sights. For some of the posters, it was before their procedure: “...woke up early and went for a tour of the old city, [Topkapi] Museum, blue mosque, and basically walked about.” For others, their sightseeing occurred after their procedures: “After the medical dressing and cleaning, I wore my hat and went for Istanbul city tour.”

##### Perceptions of Physicians

Overwhelmingly, the authors mentioned their physicians in their postings (21/36, 58%) and of those who did, most of this feedback was positive (19/21, 91%). Most postings included the physicians by name (19/36, 53%) and many (14/36, 39%) mentioned the expertise of the physician:

Dr [name] was very friendly while informing me about the operation.

I deeply felt his self-confidence during the consultation.

He seems to do a lot of conferences, which is a good thing, I think, and has contributed to at least 2 of the papers being presented at this conference. He’s keeping totally up to date on the progress in stem cell research, actively participates in cutting-edge research, and quite obviously uses his knowledge to treat his patients.

Many posters (13/36, 36%) were also appreciative of the care and professionalism provided to them by their physicians.

##### Follow-Up

Several (5/36, 14%) posters mentioned the follow-up from their providers in Turkey:

I can reach the doctor with my questions by phone. I have also emailed them and got a reply within the same day.

The posters noted that they received follow-up via email and one poster mentioned their physician’s availability via Skype:

We have stayed in contact with Dr [name] at [facility] in [town] Turkey—email and Skype are wonderful ways to maintain communications, and it has been a real treat to actually see him and his office assistant and [name], our international rep from the medical center.

##### Impression of Facilities

Many (7/36, 19%) posters noted the cleanliness of the facilities and some (3/36, 8%) commented on the surroundings and up-to-date equipment:

The hospital is bright and modern and clean.

The private hospital where I got the operation was very pleasing with its full nursing facilities and advanced medical care units.

##### Value

Many of the posters (23/36, 64%) noted that they felt that they “got their money worth” and were pleased with the value of the investment they made in their procedure(s): “Anyone needing hair transplants should think of Istanbul as the place; people and service are fine and at €1 a hair, it’s incredible value.” One couple seeking fertility treatment returned for a second round after the first in vitro fertilization in Turkey was unsuccessful.

##### Facilitators

Health travel facilitators were often mentioned by name (7/36, 19%) and evaluated by some for their assistance and responsiveness during the posters time in Turkey. These individuals greeted the posters at the airport and provided support during the stay and also facilitated site seeing etc: “When I arrived at the airport in Turkey, Dr [name]’s English translator and the German translator were waiting for me.”

Some (4/36, 11%) posters noted that the facilitators provided a direct contact number and 2 posters noted that they also received a phone subscriber identity module (SIM) card or telephone to aid in communication. “They took care of the check-in and also gave me a prepaid SIM card in case I had to contact them for any reason.”

#### Negative Factors

##### Overview

Two posters (2/12, 17%) were not satisfied with their hair growth after transplants. Several posters (7/36, 19%) noted some areas of dissatisfaction with their experience in Turkey and identified opportunities for improvements. Overwhelmingly, these negative posts focused on issues with communication and the lack of ability to speak to their caregivers in English. Food, transportation, and responsiveness were also mentioned as opportunities for improvement and several gaps in service were noted by posters who documented these details of their experiences: “I contacted [the provider] but my query was ignored—I did not get any feedback.”

##### Communication

Although many posters (8/36, 22%) described lengthy communications with their physician or the physician’s ability to speak to them in English, many posters (7/36, 19%) noted the challenges of communicating with the staff and support team members:

Language was a problem as we are from an English-speaking country, but did get along with a lot of hand language.

Whilst Dr [name] speaks English, most of the nursing staff did not, so I got by with sign language and a few words.

Several posters (4/36, 11%) noted an inability to communicate with the staff, such as nurses, and struggled to make their needs known when dealing with clinical needs: “Today’s challenge: How does one communicate, ‘I’m constipated’ with folks who don’t understand English?”

Challenges with others, such as taxi drivers, were also noted:

As we climbed in the taxi to take us home, I told the driver the name of our hotel. He said “okay,” and we were off. About halfway there he took a wrong turn (I don’t know many streets, in Istanbul, but I knew this one). A minute later, he pulled up to the wrong hotel. We said, “uhhh, this isn’t it.” He took us to one with a similar name. After that he gave us a pretty big lecture in Turkish about always getting a hotel card when we leave.

##### Food

Many of the posters who wrote about food (3/5, 60%) mentioned their challenges with having the types of food they were used to having at home: “Fruit has been not available since I got here, so bought a large bunch of grapes on my way back to the apartment.”

Those who wrote about the food served to them during their hospitalizations shared their feedback on items they were served during their stay: “Afterwards, I did manage to sleep until around 6:30 am when they brought me my breakfast—a light salad with some goat’s cheese and olives (yuck) and a cup of black tea.”

One poster who raved about her care and outcome noted the only issues she had with her stay:

Only complaint is the food SUCKED. I was only given soup for 2 days and consistency was like baby food. I had the lady helping me get some instant soup at the supermarket, much better!!

##### Transportation

Many of the posters who wrote about their experiences noted their perception of the local traffic (3/10, 33%) “The drive to the hospital was a little intense, in Turkey they are not good with following the traffic laws, so everyone seems to do what they want, a little scary.” Traffic was a barrier to one poster who was considering seeing more of the sights in Istanbul: “Traffic in Turkey is like nothing we have ever experienced, instead of trying to find a taxi and having a stressful drive to the sights, and not being sure what is and isn’t open on Sunday, staying in seemed to be the logical choice.” Two of the posters from the United States noted the multiple flights they took to get to Turkey and the challenges of traveling this distance.

##### Impressions of the People

Although most comments about the staff and general population were positive (5/7, 71%), some (2/7, 29%) employee behaviors observed by the posters were less than positive:

Monday morning, I was looking out the window as folks were coming in to work. Lots of cigarette smokers here and it’s not uncommon to see them outside the entrances to the hospital. It didn’t take me long, though, to figure out this guy was smoking more than a cigarette. One toke over the line, sweet Jesus...

Other negative touch points with individuals involved in the treatment were also noted:

My dealings were mostly with another rep, [name], who is obviously very busy juggling many patient/doctor schedules at any one time. However, maybe it was just me, but I felt my interactions with her were sometimes a little “edgy” and I was not always happy when what I considered were legitimate doubts or concerns were met with what appeared to be impatience and touchiness. Maybe it’s a cultural thing, but it wasn’t what I am used to by way of customer care, and I didn’t find it very reassuring at all.

##### Responsiveness

For the most part, posters who mentioned communicating with the facilities or providers were satisfied with their responses (6/9, 67%). However, one person who was dissatisfied with the outcome of his hair transplant noted that he repeatedly tried to get a response from the provider, but needed several attempts before he got a satisfactory response. Another poster noted that he attempted to reach an organization to arrange a health physical but did not receive a reply: “I contacted [facility] first but they neglected to return my call.”

##### Unexpected Service Gaps

Several posters (4/36, 11%) noted omissions in service or occurrences of incidents they did not expect. One patient arrived at the clinic expecting to be treated by a specific physician, only to be assigned an alternative physician:

When I arrived to the clinic on the day of my surgery, I learned that Dr [Name] cannot perform FUE [follicular unit extraction] surgeries and even more she was out of the country...and instead it will be done by a different surgeon, Dr [name].

One of the posters shared that she incurred unanticipated costs associated with her treatment:

We dealt with some frustrations regarding procuring medications once she was released as an in-patient. Unlike US hospitals, which often have pharmacies where patients can buy their drugs, in Turkey, it’s illegal for hospital pharmacies to “sell” drugs to those who are not inpatients. So we ended up going to the “outside” pharmacy for her meds for this week and found ourselves unexpectedly paying a lot of money for a very expensive drug that we thought was going to be covered in the pretransplant costs that have already been paid for.

One poster and her partner’s summary of their final hours in Turkey revealed her challenges as she tried to get to her flight:

We waited at the entrance to the airport for about a half hour while our driver went to get a wheelchair for [name]. He came back empty handed, which meant we needed to load all the luggage on one cart, and push it through security before we could get to a check-in counter.

Three of the posters mentioned that they were frustrated with having only a single English-speaking television channel and one mentioned being unable to play her US DVDs in the DVD player. A poster who stayed for an extended hospitalization noted an inability to do her laundry on-site and having to send items out for cleaning.

##### Other Negative Factors

One of the posters went to into great detail about her frustrations with getting mail sent to her in Turkey. She notes the post office demanding payment to secure a package:

The Turkish mail system doesn’t have a great reputation, so our own postal system loses control once it leaves the US.

In addition, one poster noted that when out and about in the outskirts of Istanbul, the poster noted their thoughts on air pollution and suggested to readers to avoid going outside:

Not great air quality here because of the factories so close by so going out walking too much isn’t a great idea.

The partner of the patient who stayed over 2 months in Turkey noted that after a few days her blog was censored and she had to email her posts to a friend to post from the United States:

This blog has been censored by the Turkish government so, being situated in Turkey, I can’t access it. So I’m going to try an end run by writing and then forwarding to someone in the US who can upload postings.

## Discussion

### Principal Findings

This analysis of online narratives provides significant insights into experiences of health travelers to Turkey. This study provides information from 36 individuals who posted their characteristics, the factors that drove them to leave their home counties for care, and what attracted them to seek care in Turkey. Details from the posters on the outcomes of their procedures and their satisfaction with their experiences in Turkey provide an understanding of both positive and negative factors influencing their perception of health travel to Turkey. Overall, these insights also provide individuals exploring options for health care abroad with information about others’ experiences as health travelers and may aid in the decision process of those seeking care in another country. Marketers of health travel may benefit from our analysis as they develop strategies to address the influence that the insights and opinions of current health travelers have on individuals considering future travel for care. As consumers of health care seek information on treatment options and outcomes, online outcome data and reviews may also influence their decisions of care choices.

This purposeful sample of 36 individual posters of narratives mirrors the size of samples analyzed by other researchers [42,49]. After careful reading and rereading of the narratives, we felt confident that the sample contained posts by actual health travelers who went abroad to Turkey for care and those entries by medical tourism promotional sites or health care providers were eliminated. Many of these narratives provided rich insights into the rationale for traveling to Turkey for care as well as detailed feedback regarding their thoughts and perceptions before, during, and after treatment. As noted by Seale et al [55], the narratives in the study provided a great amount of detail about the experience, including technical and intimate details and observations.

Posters of all ages traveled from many countries across the globe seeking care in Turkey for procedures that were unavailable or unaffordable in their home country, or not covered by insurance. This finding aligned with other studies on the drivers of patients looking for health care abroad [23,56,57]. Although wait time for procedures is noted as a push factor for some individuals seeking care abroad [4,10,17,58], none of the posters wrote that they were waiting for care in their home country.

Posters who traveled for care unavailable in their home countries had common frustrations with their current health care options, or lack thereof, and highlighted their desperation and the challenges faced by individuals seeking a solution for their illness or pain. Posters were often involved in lengthy research processes used to explore options for care with the Internet serving as the primary source of information and knowledge sharing.

Most of the authors of the narratives in this study were pulled to travel to Turkey for care that was less expensive than the treatment in their home country. Posters who traveled to Turkey for more affordable health care options also noted performing research before making the decision to travel outside their home country for care. Posters wrote about finding the best value through research on the Internet via reading other’s narratives and communicating with providers or health travel facilitators. As noted by Crooks et al [23], positive stories of success via online postings or word-of-mouth may serve as a motivator for individuals considering health travel. These posters’ research included information about the facility and investigating the physician’s qualifications and previous outcomes. Many of these posters mentioned the value of the treatment they received.

Over one-fifth of the posters (8/36, 22%) mentioned traveling with a spouse or family member and in some cases the spouse or family member also contributed to the narrative. These accompanying persons typically posted when the primary poster was undergoing a procedure and, in one bloggers case, after he died. Although accompanying care persons may require additional attention, resources, and have exposure to the stress of travel and caregiving, they are often key stakeholders in a health traveler’s support team [59]. Having these individuals included in the postings gave insights into the perspectives of the person who was the patient, but also their support person’s view on the experience.

These 36 narratives were written primarily from the perspective of individuals who traveled for health rather than by those interested in combining a vacation with medical care. Although travel and the ability to see the sights in Turkey was noted by some of the posters, many did not mention taking a vacation or traveling to tourist location(s) and focused their posts on the clinical and medical procedures rather than an overseas holiday.

Accreditation organizations, such as Joint Commission International, were only mentioned by 3 of the posters in parts of their narratives related to their decision-making process to choose Turkey for care. Although frequently promoted on health travel websites [28], as the number of accredited organizations focused on health travel increase, international accreditation may not be a long-term source of competitive advantage for providers seeking to care for health travelers. As accreditation becomes an expectation, creation of high levels of brand trust via marketing and loyalty through positive word-of-mouth are likely to become the key drivers of a health travel provider’s success [60,61]. In addition, although several posters mentioned their long flights to Turkey, proximity was only mentioned by one poster as a pull factor.

Most posters provided details on their procedures, outcomes, the care they received, the facilities, and their impression of Turkey, and these aspects were often imbedded into their narratives. The physicians and medical tourism facilitators were most often mentioned by the health travelers and frequently by name. Several posters noted that the accessibility of their physicians by email or phone after they returned home was important. Posters who used a health travel facilitator commented on the responsiveness and accessibility of the facilitator both before and during their visits to Turkey. These findings may be useful for future health travelers.

Few completely negative narratives were found during the extensive search for blogs and discussion board postings (3/40, 8%). Two posters noted their dissatisfaction with the outcome of their hair transplants and used their narratives as a vehicle to communicate their disappointment and frustrations.

In contrast to the physicians, the nursing staff were rarely mentioned by name and many posters shared their frustrations with being unable to communicate with the individuals directly responsible for their day-to-day care. The inability to make basic needs known to the staff, such as requests for water and help with elimination, has both patient safety and satisfaction implications that need to be addressed.

Opportunities also exist to improve various other touch points in the care of international patients in Turkey. These include improving the perception of the food served, transportation providers (especially taxis), and addressing unanticipated service gaps, such as an inability to find English-language television programs and lack of laundry services. Food was a dissatisfying element for several posters who mentioned their meals in their narratives. There is a need to address the cultural food preferences of health travelers and provide a more customized approach to menus and meals especially in the hospital setting.

Communication challenges with service personnel in Turkey, such as taxi drivers or merchants, were also noted. In one case, the poster articulated that she deferred sightseeing during her stay because of concerns about using a taxi in Istanbul. Posters also mentioned several incidents in which staff or other service providers demonstrated unprofessional behaviors. In addition, one poster noted the blocking of her blog during her stay in Turkey which resulted in her creating a work-around to be able to continue to communicate her experiences during hospitalization. The negative perceptions of government entities, such as the postal service, Internet censorship, or air quality in the city of care, although possibly isolated incidents, are additional opportunities for improvement.

Much like the growing body of patients who are sharing their experiences via Facebook and other sites, [62] these health travelers to Turkey have embraced the use of blogs, forum postings, and discussion boards to highlight their experiences, often in great detail. This research has demonstrated that analysis of online narratives provides a comprehensive review and insights into the experiences of health travelers to Turkey.

### Limitations

Although a number of processes were put in place to ensure the authenticity of the narratives, it was impossible to guarantee that all the narratives in this study were trustworthy. The narratives may have included fictitious data and there was no way for the researchers to validate most of the self-reported information [48]. In addition, although extensive focus was taken to ensure the narratives were genuine and nonpromotional in nature, it was impossible for the researchers to ensure that the blogs and forum posts reviewed were not created at the urging of a provider or heath travel facilitator. However, many posters shared both positive and less-than-positive experiences in their narratives and often provided both criticism and positive feedback in the same post. In addition, the posters did not always note their gender, country of origin, or other demographic information. However, anonymity may have also encouraged people to share more online and discuss sensitive topics and issues [51] making the 36 narratives a robust source of information. The narratives lacked examples of any orthopedic cases, such as individuals seeking knee or hip replacements, or any individuals who wrote that they were waiting for care in their home country.

Although the number of people with access to the Internet and the ability to post has increased worldwide [63], the sample did not include individuals who did not chose to create online narratives about their experiences [64] or those who lacked access to a computer or smartphone; thus, they may not be representative of the general population [55]. In addition, using the Internet as a source excludes the ability to obtain the benefits of the spoken word, such as inflection and other nuances, as well as visual cues from personal interviews [55]. The study also excludes other sources of patient feedback, such as formal surveys and word-of mouth feedback [60]

Our research was limited to the English language only. The majority of foreigners seeking and receiving health care in Turkey are from countries where English is not the main language [35]. Therefore, our results may not represent the complete spectrum of health travel experiences in Turkey. More specifically, culturally biased experiences, such as language, overall communication, and food, may not apply to all health travelers visiting Turkey.

### Conclusions

This research is believed to be the first of its kind in its approach to analyzing the online narratives of health travelers. This analysis provides an understanding of the insights of health travelers through the words of actual health travelers. The findings of this research expands the body of knowledge in medical tourism as well as serves as a platform for further qualitative and quantitative research on health travelers’ experiences.

The nonintrusive approach of the methodology used has provided candid insights into the experiences of health travelers. As transparency in health care increases and patient satisfaction data and feedback becomes more publicly available, this methodology could be applied to study other patient experiences in various health care settings.

As an increasingly important destination for health travelers, positive attributes about Turkey include the expertise and responsiveness of physicians, clinical facilities, overall satisfaction with the outcome of the procedure, and overall impressions of Turkey. Negative attributes include challenges with communication with the nonphysician staff including nurses and assistants, food, traffic and several service gaps.

Providers of international patient care may use patient experience research in positioning their services and in the development of patient care protocols for their health travelers. Additionally, hospitals may integrate patient experience research discoveries into their employee recruitment and training programs. In the future, health administration programs may reference expanded outcomes of this research as they evaluate their curricula and may decide to include additional classes on health travelers and their experiences. In the long term, this research may serve as a platform for the development of an international forum of health traveler experiences

## Abbreviations

FUE: follicular unit extraction
SIM: subscriber identity module
